# GTQC: Automated Genotyping Array Quality Control and Report

**DOI:** 10.7150/jgen.69860

**Published:** 2022-02-14

**Authors:** Shilin Zhao, Limin Jiang, Hui Yu, Yan Guo

**Affiliations:** 1 Department of Biostatistics, Vanderbilt University Medical Center, Nashville, TN.; 2 Department Internal Medicine, University of New Mexico, Comprehensive Cancer Center, Albuquerque, NM.

**Keywords:** genotyping, quality control, microarray

## Abstract

Genotyping array is the most economical approach for conducting large-scale genome-wide genetic association studies. Thorough quality control is key to generating high integrity genotyping data and robust results. Quality control of genotyping array is generally a complicated process, as it requires intensive manual labor in implementing the established protocols and curating a comprehensive quality report. There is an urgent need to reduce manual intervention via an automated quality control process. Based on previously established protocols and strategies, we developed an R package GTQC (GenoTyping Quality Control) to automate a majority of the quality control steps for general array genotyping data. GTQC covers a comprehensive spectrum of genotype data quality metrics and produces a detailed HTML report comprising tables and figures. Here, we describe the concepts underpinning GTQC and demonstrate its effectiveness using a real genotyping dataset. R package GTQC streamlines a majority of the quality control steps and produces a detailed HTML report on a plethora of quality control metrics, thus enabling a swift and rigorous data quality inspection prior to downstream GWAS and related analyses. By significantly cutting down on the time on genotyping quality control procedures, GTQC ensures maximum utilization of available resources and minimizes waste and inefficient allocation of manual efforts. GTQC tool can be accessed at https://github.com/slzhao/GTQC.

## Introduction

Genotyping arrays are microarrays that exploit the DNA complementary hybridization principle to detect single nucleotide polymorphisms (SNPs) in human samples. Genotyping arrays are typically used for investigating known disease related variants in the human genome, and they have been the driving force of Genome-Wide Association Studies (GWAS) for the last 15 years. The evolution of genotyping arrays can be divided into two phases: before and after the introduction of High-Throughput Sequencing (HTS) technology. Before HTS was introduced and became popular, genotyping arrays existed as the only option to screen for variants on a genome-wide scale. After the recent boom of HTS, genotyping arrays remained a competitive alternative to HTS, due to the customizable array designs and the dramatically lowered assay cost. At ~1/20 cost compared to whole genome sequencing, the genotyping array is still a preferential platform for large scale GWASs, which typically involve thousands of samples per study and thus take account of the assay cost as a crucial factor. Furthermore, genotyping data has been used far beyond the traditional GWAS analysis. For example, recent advancements in utilization and curation of expression quantitative trait loci 1-4 and methylation quantitative trait loci 5 show that SNPs are associated with the abundance of transcriptome and methylation. Genotyping data can also be used to construct polygenic scores 6 in Mendelian randomization studies 7, reconstruct distant pedigree 8, measure the genome-wide autozygosity with either run of homozygosity 9 or Heterozygosity Ratio 10, 11, estimate mitochondria heteroplasmy 12, and infer mitochondria copy number 13. Conventional and alternative applications of genotyping data result in thousands of array-based studies each year consistently.

Quality control appears as one of the most important steps in genotyping data workflow, as it roughly takes half of the overall processing time 14 and the time duration often surpasses that required for quality control of HTS data. Quality control procedures, especially for the market-dominating Illumina genotyping arrays, have been extensively described and are coming into a consensus. In 2013, Grove et al. described the best practices adopted by the CHARGE consortium, which used the Illumina Human Exome Bead Chip 15. One year later, a detailed protocol for the quality control of the same Human Exome Bead Chip was published 14, introducing scripts written in the combination of three languages (R, Perl, Shell). In 2017, Zhao et al. summarized quality control strategies for Illumina Human Exome Bead Chip and extended them to all types of Illumina genotyping arrays 16. Also in 2017, Wang et al. pushed one step further by introducing a quality control tool StrandScript, which particularly focuses on correcting strand errors in Illumina genotyping array 17. While the recent development of quality control strategies mostly centered on Illumina's genotyping technology, researchers also examined Affymetrix (ThermoFisher) Genome Wide Human SNP Array 6.0 genotyping data, such as those released from The Cancer Genome Atlas (TCGA). Various quality issues have been identified in public genotyping datasets 18. Therefore, it is imperative to integrate the recently proposed strategies and perspectives into a set of universal quality control protocols, and such a protocol should ideally be applicable to general genotyping platforms regardless of the array manufacturers.

Even though a multitude of insightful quality control strategies has been released, a satisfactory package is yet to be developed to take charge of the crucial quality control component of the genotyping data workflow. In the field, the existing protocols are composed of written recommendations and fragmented code chunks. Application of these established protocols requires extensive manual effort to tweak each step and then to string the outputs of discrete steps or multiple trials to a relatively comprehensive report. An automated quality control program not only significantly reduces the overall processing time for genotyping array data, but also dramatically suppresses random mistakes arising from frequent manual intervention. With the practical demand in mind, we designed and developed GTQC (abbreviated from “GenoTyping Quality Control”), an R package that automates the genotyping array quality control process for general genotyping data.

## Materials and methods

We developed the genotyping quality control software GTQC purely in the R language and made it encompass the quality control strategies we established previously 14, 16. The mandatory input format of GTQC is the standard PLINK file 19, a universal format of genotyping data. GTQC scrutinizes the genotyping data quality from the following aspects: Sample Call Rate, SNP Call Rate, Minor Allele Frequency (MAF), gender error, Heterozygosity Ratio, Hardy-Weinberg equilibrium (HWE), race error, and SNP consistencies between duplicated samples or duplicated SNPs. GTQC was developed in a component-based design, meaning that we allocated one independent component of the software to examine one aspect of the genotyping data. All components can be run independently, with their specific parameters controlled at the interface of the main R session. GTQC produces a final quality report on all covered quality metrics for an overall assessment. To demonstrate the utility of GTQC, we downloaded two sets of SNP data from public sources. The first dataset contains 306 subjects (151 Asians, 14 Blacks, 134 Caucasians, 2 American Indians, and 5 of unknown race) of the liver hepatocellular carcinoma (LIHC) cohort from TCGA. This dataset was genotyped with the Affymetrix Genome-wide Human SNP Array 6.0, which contains 934,968 SNPs. Additionally, SNP data of 2,504 subjects from The International Genome Sample Resources (IGSR), formerly known as the 1000 Genome Project, were also obtained for comparison purposes. The IGSR SNP dataset was generated not exclusively by genotyping; rather, it was created in a combination of genotyping, sequencing, and imputation.

## Results and Discussion

**Figure 1:**
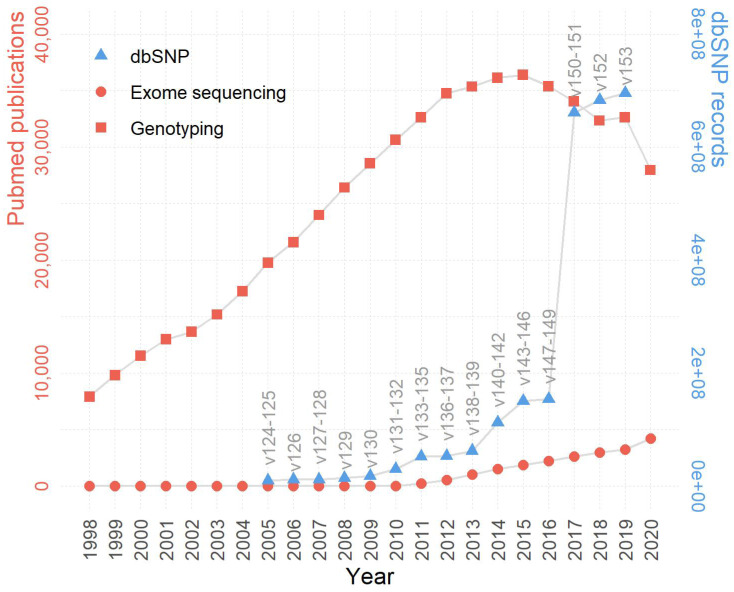
** The trends of genotype-centric studies/data in GWAS and post-GWAS era (1998-2020).** Despite a declining trend past the peak year 2015, the genotyping array based publications still largely surpass exome sequencing based publications in quantity. The growth of dbSNP is in line with the growth of genotyping and exome sequencing publications. The left y-axis denotes the number of publications (red), the right y-axis denotes the number of SNPs in dbSNP (blue), and the x-axis denotes the year.

While the HTS technology is generally considered superior to microarrays, genotyping arrays managed to maintain a substantial influence within the biomedical research field. This lasting popularity of genotyping arrays can be best represented by the immense publications based on the very technology. According to PubMed publication records, the past two decades have witnessed much more publications based on genotyping array data than those based on exome sequencing, and the trend persisted even after the recent wide adoption of HTS (Figure 1). For example, in 2020, there were roughly six times more manuscripts published using genotyping array data than using exome sequencing data. The substantially higher number of publications associated with genotyping arrays may be attributed to the more affordable price, a large amount of accumulated legacy genotyping data, and divergent applications of genotyping array data 20.

**Figure 2:**
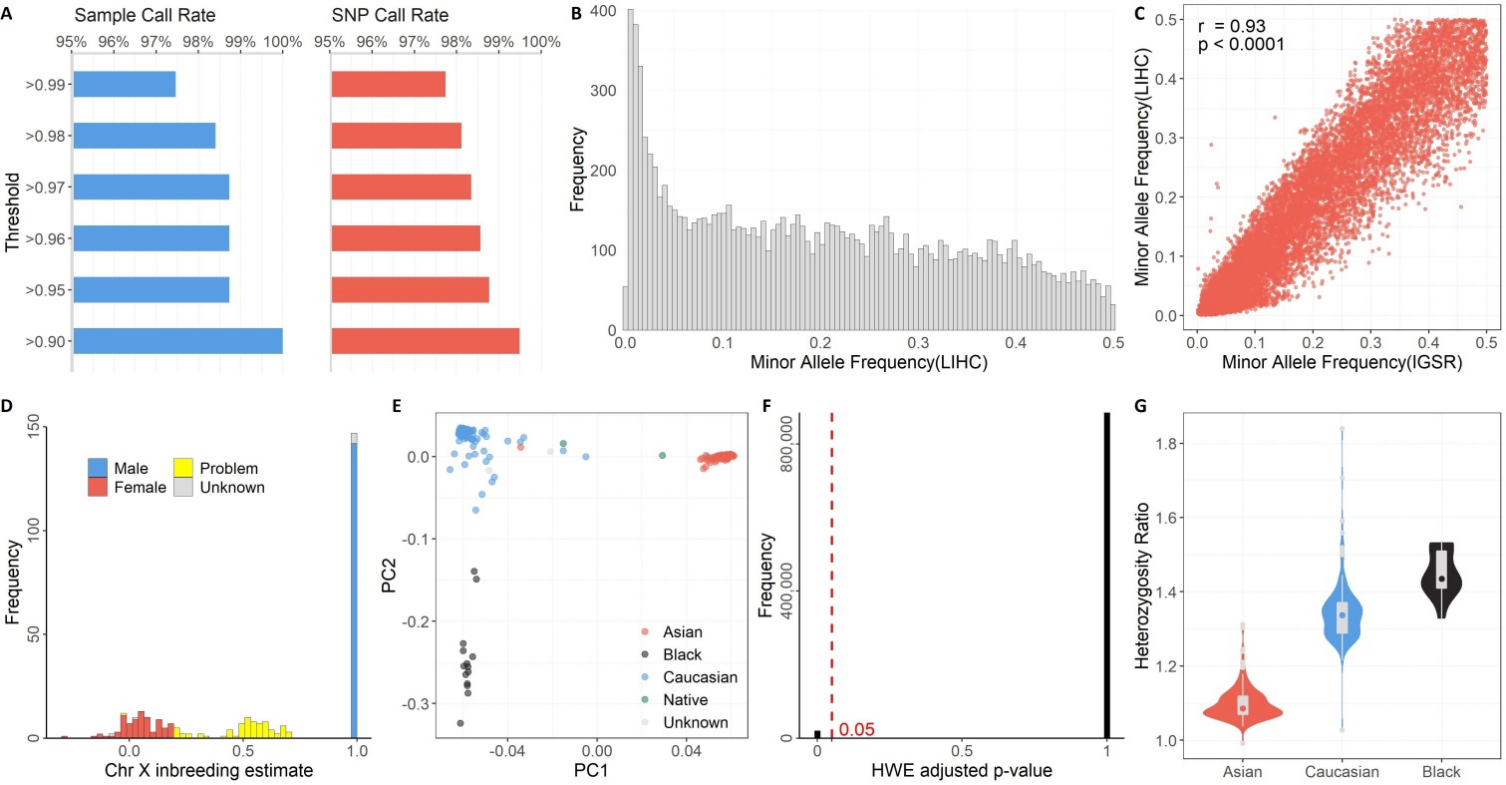
** Quality control results generated by GTQC on TCGA's LIHC genotyping dataset. A.** Summary of Sample Call Rate and SNP Call Rate at varied levels. **B.** Histogram of MAF. C. Scatter plot of MAF between TCGA's LIHC cohort and IGSR project, with only Asian subjects included. Each dot denotes the paired MAF values for a common SNP from the two cohorts. A high correlation indicates better data quality. **D.** Histogram of chromosome X inbreeding estimate. This is the result of GTQC's gender check of the example dataset. The gender check results help identify mislabeled gender information and recover gender for missing data. Alleged female cases with >0.2 inbreeding coefficient and alleged male cases with <0.98 inbreeding coefficient are flagged as problematic gender (yellow). **E.** Scatter plot of PC1 and PC2 from PC analysis. Race clusters can be clearly identified within the plot. Certain samples located between clusters suggest suspicious hybrids. **F.** Histogram of adjusted p-values from HWE check. The red dotted line denotes the threshold for statistical significance (0.05). **G.** Box and violin plots of Heterozygosity Ratios stratified by race. Due to the limited sample size, American Indians were not plotted. Subjects with unknown race information were not included either. The ethnic disparity in Heterozygosity Ratio can be seen. Asians show the lowest ratio values while black people show the highest values.

The discrete components of GTQC are aimed at the following quality control metrics: Sample Call Rate, SNP call rate, MAF, gender check, race check, HWE, and Heterozygosity Ratio. We used the SNP data from TCGA's LIHC cohort as an example to demonstrate the utility of GTQC. The representative figure results on various quality control aspects are demonstrated in Figure 2.

Defined with respect to a sample, Sample Call Rate is formulated as the percentage of SNPs called in the particular sample over the union set of SNPs called from all samples. Defined with respect to a SNP, SNP Call Rate is formulated as the percentage of SNP-presenting samples over all samples of the study cohort. It was previously recommended that 98% and 95% could be used as threshold values for Sample Call Rate and SNP Call Rate, respectively 14. In our example analyses of the TCGA LIHC cohort, all samples reached a 90% Sample Call Rate, and more than 99% of SNPs had a SNP Call Rate of 90% or higher (Figure 2A).

Unlike Sample Call Rate and SNP Call Rate, MAF cannot be derived from a single sample; rather, it is defined with respect to a subject cohort or a population. Regarding the precise chromosome locus bearing a SNP, MAF is computed as the instances of minor alleles divided by the instances of all alleles, with “instances” referring to the whole cohort. Common SNP arrays typically include SNPs with MAF >0.2 in a population. In research practice, MAF is often used as a rudimentary filter to select a specific set of SNPs by rarity. As an illustrative example, we employed GTQC to calculate MAF values for all SNPs on chromosome 21 (Figure 2B). A good practice is to compare the posterior MAF with a reference MAF established in a prior benchmark SNP dataset, such as the IGSR Project. According to our analysis, 11,883 SNPs are shared between the example dataset and IGSR dataset, and these common SNPs showed a substantial consistency in MAF between LIHC and IGSR cohorts (Pearson correlation coefficient = 0.93, p < 0.0001) (Figure 2C).

Self-reporting phenotypic variables gender and race are subject to data input errors and social-psychological confusion. Checking consistency between the reported gender and the genotyped gender provides crucial information on data integrity. Chromosomes 1 to 22 are diploid, meaning they have two copies for each SNP, either identical or variant. Besides the autosomal chromosomes, there are two sex chromosomes: X and Y. Females have two copies of chromosome X, making them diploid; males have an X chromosome and a Y chromosome. The different configuration of sex chromosomes distinguishes the males from the females genetically. The quality assessment of SNPs on a sex chromosome (X or Y) must be performed within a homogeneous gender sub-population. It is worth noting that Pseudo-Autosomal Regions (PAR) are existent on sex chromosomes. PARs are homologous regions that result from the pairing and recombining of chromosomes X and Y during meiosis in evolution. Often the so-called “chromosome XY” is tagged with SNPs on PARs, but, in some arrays, the PAR SNPs are simply labeled with Chromosome X. Thus far, there have been three PARs identified 21, 22. SNPs in PARs should be treated as diploid and thus handled in the same way as common autosome SNPs, rather than as sex chromosome SNPs. SNPs on chromosome X can be exploited by GTQC to estimate the gender of the subject. Problematic SNPs on sex chromosomes that suggest suspicious gender self-reporting are identified by GTQC (Figure 2D). Of the 306 subjects, 209 are reported males, 90 are reported females and 7 are of unknown sex. Males should have chromosome X inbreeding estimate close to one (expected range: 0.98 - 1), while females should have chromosome X inbreeding estimate close to zero (expected range: -0.3 - 0.2). According to GTQC gender checking, of the seven gender-unknown subjects, two are females and five are males. The chromosome X inbreeding estimates for 74 subjects fall out of the expected ranges, indicating problematic data quality or mislabeled gender information. For example, in our analysis results for the TCGA LIHC cohort, one TCGA subject (TCGA-GJ-A9DB) is labeled as male, but the chromosome X inbreeding estimate of -0.02 suggests female.

Like gender, race is another phenotypical variable that can be complicated with self-identification bias or data input error. Researchers often perform principal component (PC) analysis on ancestry informative markers (AIMs) to yield a genetically determined race and further use the genetically determined race as a surrogate for the self-reported race. AIMs are SNPs that exhibit considerable disparate allele frequencies between populations of different ethnicities. Most genotyping arrays encompass a number of AIMs. GTQC performs PC analysis and reports the first and second most accountable principal components, termed PC1 and PC2, respectively. In their association models, genetic association studies often adjust for the first few PC instead of the actual race, because the PCs can capture the intrinsic genetic difference more accurately even within a population ostensibly of a same race 23. Multiple races of distinct genetic profiles typically form segregated clusters on the PC1-vs-PC2 scatter plot (Figure 2E), where obvious outlier samples or hybrid race samples can be visually discerned. For example, in our analysis results for the TCGA LIHC cohort, one TCGA subject (TCGA-G3-A5SI) is labeled as Asian, but it is closer to the Caucasian cluster rather than the Asian cluster. This suggests that either this sample's race is mislabeled, or this subject is of the hybrid race between Caucasian and Asian.

The HWE principle asserts that, in a randomly mating population devoid of genetic drift or other external disturbance, the genotype distribution among the population remains an equilibrium state in a long, cross-generation timespan. As indicated by the aforesaid constraints, when the equilibrium state is disturbed, it usually suggests unusual events including population stratification, genotyping errors, or association to the very trait under study 24, 25. Within the quality control context, deviation from HWE is frequently regarded as a warning sign of genotyping errors in large GWAS set upon unrelated individuals 26, 27. While it is not uncommon for GWAS to encompass diverse populations, HWE test should be conducted race by race, because there is not an expected genotyping equilibrium across population boundaries. Furthermore, many genotyping datasets were generated in a case-control study design, in which case the HWE test should use only the control samples - as we put above, some diseases can cause deviation from HWE at the disease associated loci 28. GTQC reports the distribution of Bonferroni-adjusted HWE p-values (Figure 2F). In our analysis results for the TCGA LIHC cohort, 6.24% of SNP had an adjusted p-value < 0.05, indicating potential violation of HWE. These SNPs should be either removed from downstream analysis or flagged for further quality check.

For a genotyping dataset that involved a homogeneous sample population and covered a large number of SNPs, computing the Heterozygosity Ratio can help identify problematic SNPs. Heterozygosity Ratio is computed for a subject as the ratio between the number of heterozygous SNPs and the number of non-reference homozygous SNPs. The Heterozygosity Ratio was originally proposed by our group as a quality control parameter for SNP data, and we proved that it has a theoretically expected value of two 29. An empirical data study showed that the Heterozygosity Ratio is dependent on race 30: only African ancestry individuals approximate the theoretical value of two, whereas populations of other races fall far below two. Such a disparity in Heterozygosity Ratio is reproduced in our analysis of the TCGA LIHC dataset (Figure 2G). Specifically, exceedingly high heterozygosity may indicate sample contamination, and exceedingly low heterozygosity may indicate inbreeding.

GTQC is also capable of computing consistency between duplicated samples and duplicated SNPs. International GWAS and alike projects almost always include external control samples, such as those curated by HapMap 31 or 1000 Genome Project 32. Internal duplicated samples are also commonly included in common GWAS studies. Genotype consistency among these duplicated control samples is a crucial metric to signify the quality of genotyping assay. GTQC has a specialized module to interrogate genotyping consistency based upon user-designated control samples and duplicated samples. Because of the lack of duplicated samples in our example dataset, this particular feature is not demonstrated, but readers can refer to our prior work 16 to learn about the relevant rationale and empirical parameters that GTQC adopted.

## Conclusion

High-throughput genomic technology has been the driving force for biomedical research for the last two decades. One early type of high-throughput genomic technologies are the DNA hybridization based genotyping array, which was the backbone of the GWAS era. A large amount of genotyping array data have been deposited into public genomic data repositories and are available for secondary analysis. For example, the TCGA project provides both blood and tumor genotyping data on all of their participants. As of February 2021, over 400 genotyping array datasets with millions of subjects have been curated in the most authoritative database of genotypes and phenotypes, dbGAP 33. These data have been severely underutilized. Given the increasing popularity of alternative applications of genotyping data, these data will provide substantial data mining opportunities. However, many of the publically available datasets did not go through proper quality control prior to release. For example, it has been reported TCGA's released genotyping data contains potential cross-contamination 34. In our analysis of TCGA's LIHC cohort, we also identified potential mislabeled gender and race information. Such suspicious examples call for thorough quality control on genotyping data regardless of data origin. GTQC reduces the overall genotyping array processing time by providing automated quality control analysis on genotyping data. Because the input data is PLINK format file, Genotype QC is not limited to a specific array type or an individual manufacturer. GTQC generates a final report as an excellent guide for assessing the overall genotyping quality and identifying problematic samples and SNPs.

## Abbreviations

AIM: ancestry informative marker
GTQC: genotyping quality control
GWAS: genome wide association study
HTS: high-throughput sequencing
HWE: Hardy-Weinberg equilibrium
IGSR: the international genome sample resources
LIHC: liver hepatocellular carcinoma
MAF: minor allele frequency
PC: principal component
SNP: single nucleotide polymorphism
TCGA: The Cancer Genome Atlas
